# Sequence and Copy Number Analyses of *HEXB* Gene in Patients Affected by Sandhoff Disease: Functional Characterization of 9 Novel Sequence Variants

**DOI:** 10.1371/journal.pone.0041516

**Published:** 2012-07-27

**Authors:** Stefania Zampieri, Silvia Cattarossi, Ana Maria Oller Ramirez, Camillo Rosano, Charles Marques Lourenco, Nadia Passon, Isabella Moroni, Graziella Uziel, Antonella Pettinari, Franco Stanzial, Raquel Dodelson de Kremer, Nydia Beatriz Azar, Filiz Hazan, Mirella Filocamo, Bruno Bembi, Andrea Dardis

**Affiliations:** 1 Regional Coordinator Center for Rare Diseases, University Hospital Santa Maria della Misericordia, Udine, Italy; 2 Centro de Estudio de las Metabolopatias Congénitas, CEMECO, University of Córdoba, Córdoba, Argentine; 3 Patologia Molecolare Integrata – A.O.U. IRCSS San Martino – IST, Istituto Nazionale per la Ricerca sul Cancro, Genova, Italy; 4 Medical Genetics Service, Clinics Hospital of Ribeirao Preto, University of Sao Paulo, Sao Paulo, Brazil; 5 Dipartimento di Scienze Mediche e Biologiche, Università di Udine, Udine, Italy; 6 Department of Child Neurology, Fondazione Istituto Neurologico Besta, Milan, Italy; 7 Laboratorio di Genetica Medica, Clinica Pediatrica, Ospedali Riuniti Ancona, Ancona, Italy; 8 Servizio di Consulenza Genetica, Centro Provinciale di Coordinamento della Rete delle Malattie Rare, Azienda Sanitaria dell’Alto-Adige, Bolzano, Italy; 9 Medical Faculty, Genetic Department, Izmir, Turkey; 10 U.O.S.D. Laboratorio Diagnosi Pre-Postnatale Malattie Metaboliche, Istituto G. Gaslini, Genova, Italy; Sudbury Regional Hospital, Canada

## Abstract

Sandhoff disease (SD) is a lysosomal disorder caused by mutations in the *HEXB* gene. To date, 43 mutations of *HEXB* have been described, including 3 large deletions. Here, we have characterized 14 unrelated SD patients and developed a Multiplex Ligation-dependent Probe Amplification (MLPA) assay to investigate the presence of large *HEXB* deletions. Overall, we identified 16 alleles, 9 of which were novel, including 4 sequence variation leading to aminoacid changes [c.626C>T (p.T209I), c.634C>A (p.H212N), c.926G>T (p.C309F), c.1451G>A (p.G484E)] 3 intronic mutations (c.1082+5G>A, c.1242+1G>A, c.1169+5G>A), 1 nonsense mutation c.146C>A (p.S49X) and 1 small in-frame deletion c.1260_1265delAGTTGA (p.V421_E422del). Using the new MLPA assay, 2 previously described deletions were identified. *In vitro* expression studies showed that proteins bearing aminoacid changes p.T209I and p.G484E presented a very low or absent activity, while proteins bearing the p.H212N and p.C309F changes retained a significant residual activity. The detrimental effect of the 3 novel intronic mutations on the *HEXB* mRNA processing was demonstrated using a minigene assay. Unprecedentedly, minigene studies revealed the presence of a novel alternative spliced *HEXB* mRNA variant also present in normal cells. In conclusion, we provided new insights into the molecular basis of SD and validated an MLPA assay for detecting large *HEXB* deletions.

## Introduction

Sandhoff disease (SD) [MIM:268800] is a rare neurodegenerative disorder resulting from the inability of the ß-hexosaminidase [Hex; EC: 3.2.1.52] to cleave the terminal N-acetylhexosamine residues from GM2 ganglioside [1]. Two major Hex isoenzymes exist: HexA, a heterodimer composed of α/ß subunits and HexB, a homodimer composed of ß subunits. *In vivo*, only Hex A isoenzyme is capable of degrading the GM2 ganglioside in a complex with the cofactor GM2 activator protein.

Alpha and beta subunits are encoded by the *HEXA* (MIM:606869) and *HEXB* (MIM:606873) genes, respectively. The GM2A activator protein is encoded by *GM2A* gene (MIM: 613109).

Mutations affecting *HEXA, HEXB* or *GM2A* genes result in a group of recessive disorders called GM2 gangliosidoses, characterized by the accumulation of GM2 ganglioside [1], [2].

Sandhoff disease (SD) results from mutations of the *HEXB* gene. Therefore, both HexA and HexB isoenzyme activities are reduced or absent.

The clinical phenotype varies widely from the infantile form, characterized by the early onset of a rapidly progressive neurodegenerative disease, leading to dead before the fourth year of life, to the later onset forms, a progressive neurological condition compatible with survival into childhood (subacute form) or long survival (chronic form) [1].

*HEXB* gene, mapped to chromosome 5q13 (GeneBank accession number NM_000521), spans 35–40 Kb and contains 14 exons. Up to date, only 43 *HEXB* mutations have been reported including the large common deletion of 16 kb [Human Gene Mutation Database (http://www.hgmd.org) [3]. This latter deletion, accounting for about 27% of the SD alleles among various ethnic groups [4], [5] has never been identified in Italian SD patients.

We have previously reported the study of *HEXB* gene in 12 unrelated SD patients [6]. Here, we report the molecular characterization of the *HEXB* defect in a further 14 SD unrelated patients with different geographical/ethnic background. This study comprises the functional analysis of 9 new sequence variations identified in the *HEXB* gene, including 5 missense (4 novel and 1 previously described) and 3 splicing mutations. In addition to the conventional methods, a Multiplex Ligation dependent Probe Amplification (MLPA) assay for copy number analysis of the 14 exons of *HEXB* gene has been developed to detect possible large gene deletions.

## Methods

The study was approved by the Ethic Committee of the University Hospital “Santa Maria della Misericordia”, the Institutional Ethics Committee for Health Research, CIEIS, Children’s Hospital, San Roque’s Hospital and Rawson’s Hospital, Córdoba-Argentina and the Ethic Committee of the University of Sao Paulo. Written consent was obtained from subjects or carers/guardians on the behalf of the minors involved in the study.

### Patients

Fourteen unrelated patients affected by SD with different geographical/ethnic background were included in this study. The diagnosis was suspected on the presence of neurological symptoms and was confirmed by the demonstration of reduced or absent total Hex activity in plasma, peripheral white blood cells or cultured fibroblasts. Four patients were of Italian origin, 5 were Argentineans, 2 Brazilians, 1 Turkish, 1 Bulgarian and 1 Chinese.

### Mutational Analysis of *HEXB* Gene

Genomic DNA was extracted from peripheral blood leukocytes or cultured fibroblasts with Imp DNA blood Mini Kit (Qiagen GmbH, Hilden, Germany). The exonic and flanking intronic sequences of the *HEXB* gene were amplified by PCR and analyzed by automated sequencing (ABI Prism 3500xl genetic analyzer) as previously reported [6]. Putative mutations were confirmed by sequencing duplicate PCR products and by the DNA analysis from parents and relatives whenever possible.

### Multiplex Ligation-dependent Probe Amplification (MLPA) Analysis of *HEXB* Gene

A set of 17 synthetic probes for MLPA analysis of *HEXB* gene was developed according to the guidelines available at http://www.mrc-holland.com. Thirty four gene-specific oligonucleotides were used to amplify and to detect 17 different gene-specific signals: 14 were exon-*HEXB* specific, while other3, not related to *HEXB* gene and specific for alpha-centractin (*ACTR1a)*, actin filament-associated protein (*AFAP)* and albumin *(ALB)*, were used as control for copy number variation (). Primers are listed in Table S1. MLPA analysis was carried out as described by Schouten et al [7]. Briefly, 50 ng of DNA in a volume of 4,5 µl were denatured for 5 min at 98°C. 1.5 µl of salt solution (1.5 M KCl, 300 mM Tris-HCl pH 8.5, 1 mM EDTA) and 2 µl of probe mix (1–8 fmol of each synthetic probe oligonucleotide) were added and allowed to hybridize with the DNA target at 60°C for 16 h. Ligation of annealed probes was performed in a final volume of 40 µl obtained by adding 32 µl of ligation buffer (2,6 mM MgCl2, 5 mM Tris-HCl pH 8.5, 0.013% Triton X-100), 0.2 mM NAD+(BioLabs, Milan, Italy) and 0.2 U of the enzyme Ligase-65 (MRC-Holland, Milan, Italy). The ligation mix was incubated for 15 min at 54°C. The Ligase-65 was inactivated by heating at 98°C for 5 min. PCR reaction was carried out in a mixture containing 5 µl of ligation products, 1.25 U AmpliTaq Gold (Applied Biosystems, Warrington, UK), the enzyme buffer (Tris-HCl pH8.3, 1.5 mM MgCl2, 50 mM KCl), 200 nM of forward and reverse universal primers and 200 µM of dNTPs mix (Invitrogen, Carlsbad, CA, USA) in a final volume of 25 µl. PCR was carried out under the following conditions: 95°C for 1 min, followed by 35 cycles of 30 s at 95°C, 30 s at 60°C and 1 min at 72°C. A final extension step was programmed for 20 min at 72°C.

Amplification products were identified and quantified by capillary electrophoresis on an ABI Prism 3500xl genetic analyser, using Genescan analysis software (version 4.1) (Applied Biosystems, Warrington, UK).

The relative quantification of *HEXB* copy number was obtained by dividing the height of each gene-specific peak by the sum of the 3 endogenous control peaks present in 2 copies per diploid genome. This ratio was then compared to the average ratio obtained from 8 control samples having each 2 *HEXB* gene copies. A ratio between 0.75 and 1.25 corresponds to 2 *HEXB* copy number while a ratio between 0.25 and 0.75 corresponds to 1 *HEXB* copy number.

### In Silico Analysis

To predict the potential effect of the novel mutant alleles on splicing process, the following programs were used: Maximum Entropy (ME) available at http://genes.mit.edu/burgelab/maxent/Xmaxentscan_scoreseq_acc.html and Neural Network (NN) available at http://www.fruitfly.org/seq_tools/splice.html.

### Protein Visualization and Structural Analysis

For modelling the *HEXB* missense mutations, we used the three dimensional atomic coordinates available at the Protein Data Bank (PDB) of the apo-protein and of its form bound to a known inhibitor [8] (PDB codes 1NOU and 1NOW). Visual inspection and graphical representations were then carried out using the programs Coot [9] and Chimera [10], respectively.

### *HEXB* Missense Constructs

To assess the potential impact of 5 missense *HEXB* sequence variations on Hex activity we performed *in vitro* expression experiments. The complete cDNA of *HEXB* was cloned in the pcDNA4/*myc*-His (Invitrogen, Carlsbad, CA, USA) expression vector (pcDNA4/mycHEXBN). Mutations p.T209I (pcDNA4/mycHEXB209), p.N212H (pcDNA4/mycHEXB212), p.C309F (pcDNA4/mycHEXB309), p.G484E (pcDNA4/mycHEXB484) and p.R533C (pcDNA4/mycHEXB533) were introduced in the pcDNA4/mycHEXBN construct by site directed mutagenesis using the QuikChange Site-Directed Mutagenesis Kit (Stratagene, Cedar Creek, TX, USA) according to the manufacturer’s instructions. Primers are listed in Table S2.

### Minigene Constructs

To evaluate the effect of the intronic *HEXB* mutations c.1082+5G>A, c.1169+5G>A and c.1242+1G>A on exon 8, 9 and 10 expression, normal minigenes (p.HEXB7-9 N, p.HEXB8-9 N and p.HEXB9-11 N) were prepared by cloning the genomic sequences from exons 7 to 9, 8 to10 and 9 to 11 of *HEXB*, respectively, in pcDNA3.1 (Invitrogen, Carlsbad, CA, USA). PCR amplification was performed using primers Minigene *HEXB* 7-8-9 Fw-Rv, Minigene *HEXB* 8-9-10 Fw-Rv and Minigene *HEXB* 9-10-11 Fw-Rv, listed in Table S2. The forward and reverse primers used for the amplification carried the *NotI* and *Xho* restriction site, respectively.

Mutant minigenes, p.HEXB7-9 mut, p.HEXB8-9 mut and p.HEXB9-11 mut carrying the sequences bearing the c.1082+5G>A, c.1169+5G>A and c.1242+1G>A substitutions, respectively, were prepared by site directed mutagenesis using the QuikChange Site-Directed Mutagenesis Kit (Stratagene, Cedar Creek, TX, USA) according to the manufacturer’s instructions. Primers are listed in Table S2.

### Cell Culture and Transient Transfection

Human Hek293 [11] cells were grown in DMEM high glucose medium (Gibco, Paisley, UK) supplemented with 10% (v/v) foetal bovine serum, 50 mg/ml penicillin/streptomycin and 2 mM L-glutamine (Gibco, Paisley, UK). Cells were maintained at 37°C in a humidified atmosphere enriched with 5% (v/v) CO2 and transfected with Lipofectamine 2000 (Invitrogen, Carlsbad, CA, USA) using 4 µg of total plasmid DNA Endofree purified (Qiagen GmbH, Hilden, Germany) following manufacturer’s instructions. To normalize transfection efficiency between experiments, cells were transiently cotransfected with 2 µg of a construct bearing the GFP cDNA, and the fluorescence was measured by FACS analysis.

### Minigene Splicing Assay

Hek293 cells were transfected with the normal and mutant minigene constructs. Total RNA was extracted after 48 h using TRIzol reagent (Gibco, Paisley, UK) and analyzed by RT-PCR. Reverse transcription was performed using random primers; the PCR reaction was carried out with a forward vector-specific primer (5′-AATACGACTCACTATAGGG) and the reverse primers Minigene *HEXB* 7-8-9 Rv, Minigene *HEXB* 8-9-10 Rv and Minigene *HEXB* 9-10-11 Rv (Table S2). PCR products were resolved in a 1% agarose gel and sequenced.

### Immunoprecipitation and Enzyme Activity Assay

Hek293 cells were transfected with normal and mutant myc tagged constructs. Forty eight hours after transfection, cells were washed with phosphate-buffered saline (PBS), harvested and sonicated. Protein concentration of the samples was determined by the Bradford method. Solid-state immunoprecipitation assay for Hex was performed based on the previous report [12]. Briefly, 140 µl of cell lysate corresponding to 20 µg of protein was rotated at 4°C overnight with 30 µl of protein A Sepharose beads (Amersham Pharmacia Biothech, NJ) and 1 µl of anti-myc mouse monoclonal antibody (Cell signalling, Hitchin, UK).

After centrifugation for 15 min at 12,000 g, precipitated beads were washed twice with cold PBS and assayed directly for determining Hex activity. Total Hex activity was measured using the fluorogenic substrate 4-methyllumbelliferyl-2-acetamido-2-deoxy-β-Dglucopyranoside (4MUG; Sigma, St. Louis, MO, USA). Total activity was estimated by incubation at 37°C for 15 min in the presence of 3 mM of 4MUG and 0.1 M of citrate–phosphate buffer (pH 4.6) in a total volume of 300 µl. Reactions were stopped by adding 1.5 ml of 0.2 M glycine–NaOH buffer (pH 10.6). Fluorescence excitation was conducted at 365 nm wavelength and emission was determined at 495 nm. The residual enzymatic activity was expressed as percentage of the values obtained in cells transfected with the normal construct.

### Mutation Nomenclature

All mutations are described according to the recommended nomenclature [13], [14] http://www.hgvs.org/mutnomen. Nucleotide numbers are derived from cDNA sequences (GenBank *HEXB* cDNA:NM_000521).

### Statistical Analysis

Statistical analysis was performed using Student’s t test analysis. P<0.05 was considered as statistically significant.

## Results

### Molecular Analysis of *HEXB* Gene

The molecular analysis of *HEXB* gene was performed in 14 patients affected by SD.

Table 1 shows the geographic/ethnic background, age at onset, age at last observation, total Hex activities and the 13 different genotypes identified in this series.

**Table 1:** Genotypes of SD patients analyzed in this study.

Patient	Origin	Age atonset	Age atobservation	Total Hex activity§(% of normal controls)	Genotype*
SD1	Italy	18 m	10 y^+^	2.8	**[c.626C>T(p.T209I)]+** [c.299+1471_408del2406]
SD2	Italy	4 m	3y9m^+^	11.5	[c.1303_1304delGT(p.R435fsX20)]+ **[c.926G>T(p.C309F)]**
SD3	Italy	6 m	2y6m^+^	2.6	**[c.1451G>A(p.G484E)]+**[?]
SD4	Italy	6 m	2y3m^+^	3.1	[c.448A>C(p.T150P)]+ [16Kbdel]
SD5	Argentine	4 m	2y8m^+^	1.1	[c.445+1G>A (r.0)]+[**c.1451G>A(p.G484E)]**
SD6	Argentine	4–6 m	2y11m^+^	1.3	[c.445+1G>A (r.0)]+[c.1597C>T(p.R533C)]
SD7	Argentine	4–6 m	3y4m^+^	4.2	[c.445+1G>A (r.0)]+[**c.1242+1G>A (p.K390_K414delfsX7)]**
SD8	Argentine	4–5 m	3y^+^	ND	[c.445+1G>A (r.0)]+[**c.1242+1G>A (p.K390_K414delfsX7)]**
SD9	Argentine	4–5 m	3y+	ND	**[c.1082+5G>A(p.G301_W361delfsX10)]+** [c.1601G>A(p.C534Y)]
SD10	Brazil	12 m	4y2m	2.8	**[c.1169+5G>A(p.E362_K390del)]+** [c.448A>C(p.T150P)]
SD11**	Brazil	8 m	3y9m	8.1	**[c.634C>A(p.H212N)]+ [c.634C>A(p.H212N)]**
SD12	Turkey	4m	2y +	NA	[c.1597C>T(p.R533C)]+ [c.1597C>T(p.R533C)]
SD13	Bulgaria	9 m	1y3m	1.6	**[c.146C>A(p.S49X)]+ [c.146C>A(p.S49X)]**
SD14	China	12 m	2y	15	**[c.1260_1265delAGTTGA(p.V421_E422del)]+ [c.1260_1265delAGTTGA(p.V421_E422del)]**

ND: not detectable; NA: not available. Novel mutations are indicated in bold, *RefSeq cDNA:NM_000521. For cDNA numbering +1 corresponds to the A of the first ATG translation initiation codon. RefSeq protein: NP_000512.1. **indicates parents’ consanguinity. m: month; y: years; +deceased; §Owing to the use of different assay methods and tissue samples, total Hex activity values are expressed as a percentage of average control values.

Thirteen patients presented with the severe infantile form of SD characterized by early onset, between 4–12 months of age, while one patient (SD1) presented with the subacute form of the disease. At diagnosis, all patients presented with hypotonia, psychomotor skills regression and the presence of a cherry-red spot on fundus examination, except for patient SD14 who did not have this last ocular sign. Startle reaction was reported in 11 patients (SD1 to SD11). Patients SD3, SD5-SD8, SD10- SD12 and SD14 developed seizures. Ten infantile patients died before the fourth year of life, while 3 are still alive; patient SD1, with the subacute form died at the age of 10 years (Table 1).

Most patients were of Italian and Argentinean origins, except patients SD12, SD13 and SD 14 who were of Turkish, Bulgarian and Chinese ancestry, respectively, and patients SD10 and SD11 who were of Brazilian ancestry. Parents of patients SD11 and SD14 were consanguineous. Argentinean patients SD5, SD7 and SD8 were from a geographic area in Cordoba characterized by a high SD incidence [15], while SD6 and SD9 were from Buenos Aires.

Overall, the mutational spectrum of the present series comprises 16 different alleles, including 9 previously unpublished. Among the novel alleles, 4 were sequence variations resulting in codon replacements [c.626C>T (p.T209I), c.634C>A (p.H212N), c.926G>T (p.C309F), c.1451G>A (p.G484E)]; 3 were splice site alterations (c.1082+5G>A, c.1242+1G>A, c.1169+5G>A), 1 was a nonsense mutation c.146C>A (p.S49X), and 1 was a small in-frame deletion c.1260_1265delAGTTGA (p.V421_E422del). One allele remained unknown. even after MLPA assay.

### Detection of Deletions in *HEXB* Gene through MLPA Assay

**Figure 1:**
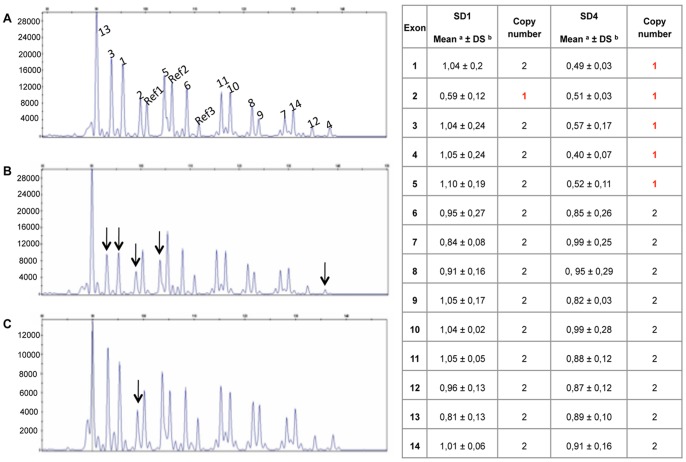
MLPA analysis of SD1 and SD4 patients. Panels A–C: Capillary electrophoresis profile of the MLPA analysis performed in a normal control (panel A) and patients SD4 (panel B) and SD1 (panel C). Each peak corresponds to the amplification of a probe specific for each exon of *HEXB* gene (numbered 1 to 14) and for 3 different reference genes (R1, R2, R3). Arrows indicate the peaks corresponding to the *HEXB* exons deleted in SD1 and SD4 patients. Panel D) Relative quantification of *HEXB* copy number was obtained by dividing the height of each gene-specific peak by the sum of the heights of 3 reference gene peaks. This ratio was then compared to the average ratio obtained from 8 control samples having each 2 *HEXB* gene copies. A ratio between 0.75 and 1.25 corresponds to 2 *HEXB* copy number while a ratio between 0.25 and 0.75 corresponds to 1 *HEXB* copy number.

In order to analyze the presence of large deletions of the *HEXB* gene non detectable by PCR and sequencing, we developed a synthetic probe set for multiplex ligation-dependent probe amplification (MLPA) analysis [7]. After optimizing the probe specificity for *HEXB* and references genes, 14 SD patients, 8 normal controls and 2 positive controls carrying two previously described deletions, c.299+1471_408del2406 (here named as Del1) [6] and the 16 kb deletion (here named as Del2) [16], respectively, were analyzed. The c.299+1471_408del2406 has been reported to remove part of intron 1 and exon 2, while the 16 Kb deletion comprises the promoter region, exons 1–5 and part of intron 5. The MLPA analysis showed the presence of a partial *HEXB* deletion affecting exon 2 in SD1 patient and a partial *HEXB* deletion affecting exons 1 to 5 in SD4 patient (Figure 1), while other SD patients showed a normal pattern (data not shown). MLPA results were also confirmed in SD1 and SD4 patients’ parents. PCR and sequencing analysis confirmed the presence of the c.299+1471_408del2406 deletion in the patient SD1 and the 16 Kb deletion in the patient SD4 (data not shown).

### Characterization of the Novel Missense Sequence Variants

Four novel missense variations were found in 6 SD families: c.626C>T (p.T209I), c.634C>A (p.H212N), c.926G>T (p.C309F), c.1451G>A (p.G484E).

The impact of these 4 aminoacid changes on protein function was evaluated by *in vitro* expression of mutant constructs in Hek293 cells and the analysis of the expressed Hex activity after immunoprecipitation. As a negative control, Hek293 cells were transfected with empty pcDNA4/*myc*-His vector. In addition, the mutation p.R533C (c.1597C>T), although no longer novel since it was reported during the preparation of the present study by Kaya et al [17], was also included in the analysis.

**Figure 2:**
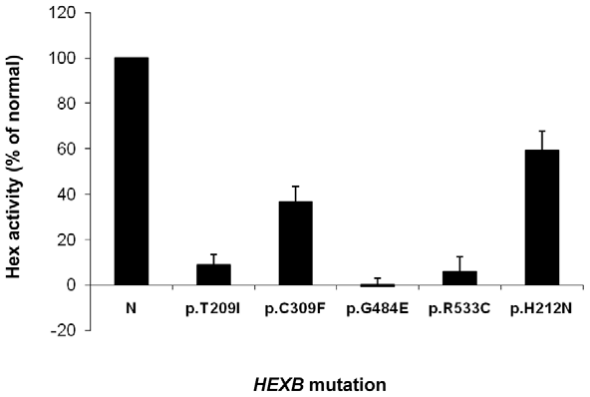
*In vitro* functional analysis of new missense sequence variations. Total Hex activity after immunoprecipitation with anti-myc antibody of HEXB missense mutant proteins expressed in Hek293cells. Results are expressed as the percentage of total Hex activity detected after immunoprecipitation of myc-tagged normal HEXB expressed in Hek293cells (N). The data are shown as mean±SD of three different experiments, each performed in duplicate. ^*^p<0.05.

Since untransfeced Hek293 cells showed a quite high level of endogenous Hex activity, we transfected them with constructs designed to generate myc tagged normal and mutant HexB proteins, which could be easily separated from the endogenous HexB by immunoprecipitation with an anti-myc antibody. Transfection of Hek293 cells with the normal *HEXB* cDNA resulted in an 8 to 10-fold increase of total Hex activity measured on the immunoprecipitated complex, compared to the activity of Hek293 cells transfected with the empty vector. No changes in the viability of transfected cells were observed. As shown in Figure 2, the *in vitro* residual activity of the 5 missense mutants was significantly lower than the activity of the normal protein. However, while p.T209I, p.G484E and p.R533C mutant variants showed a very low or absent Hex activity as compared to the normal protein, proteins bearing the p.H212N and the p.C309F aminoacid changes retained a residual activity of 59 and 36% of normal, respectively (Figure 2). In the light of these data, further experiments were planned to exclude the potential occurrence of additionally intronic mutations affecting the mRNA splicing process. In the case of p.C309F, found in heterozygosis with the severe c.1303_1304delGT (p.R435fsX20) mutation [18], the RT-PCR analysis of the *HEXB* mRNA in fibroblasts from patient SD2 showed the presence of a single product, which was confirmed by sequencing analysis as corresponding to the normal spliced *HEXB* transcript carrying the p.C309F mutation. This excluded the potential coexistence of other mutations on the same allele. Unfortunately, the mRNA of the patient SD12, homozygous for p.H212N, was not available to exclude the potential occurrence of a second *in cis* mutation. Although biochemical data were suggestive of Sandhoff disease, other clinical forms of GM2 Gangliosidosis (Tay-Sachs and GM2-gangliosidosis, AB variant) were considered and *HEXA* and *GM2A* genes were analysed, excluding a potential clinical misdiagnosis.

**Figure 3:**
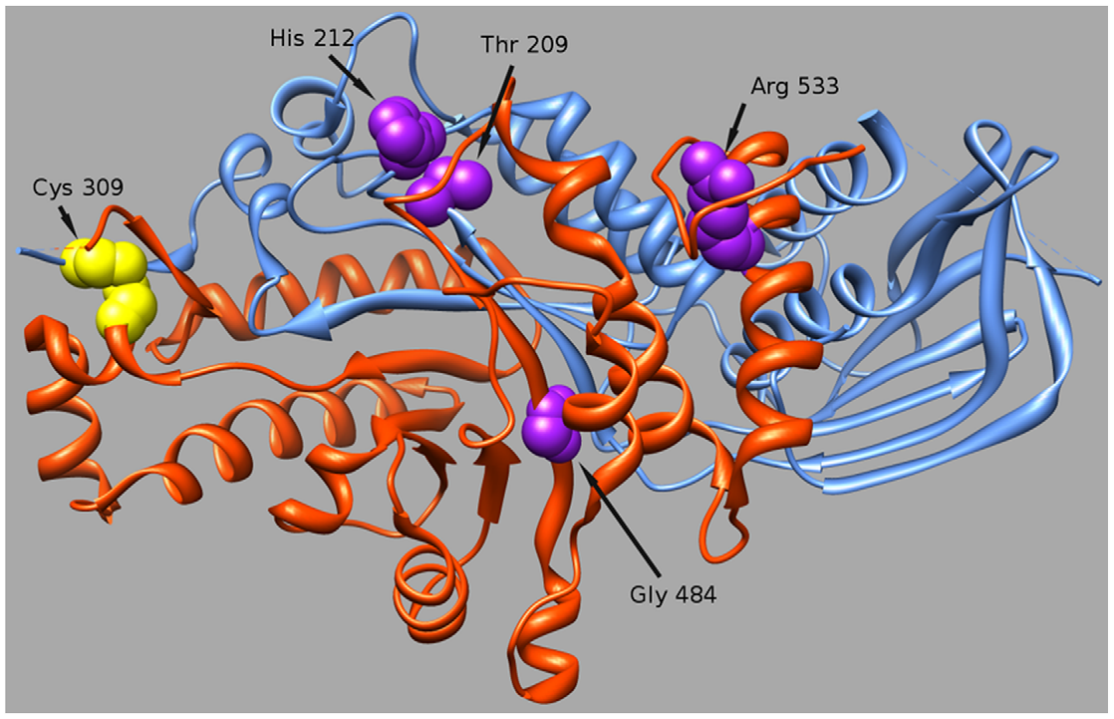
Location of HEXB mutations on the 3D structural model. Chains A and B are drawn as orange and blue ribbons respectively. Positions of mutations are indicated by arrows and the residues represented by spaced-filled spheres.

To shed further light on the possible consequences of these aminoacid changes at protein level, we visualized the atomic structure of HexB determined by X-ray Crystallography (Figure 3).

p.T209I: the structural analysis of the mutation predicted that the replacement of a hydrophilic residue (T) with the hydrophobic isoleucine (I), occurring in the proximity of a residue (R211) know to be involved in substrate binding, may lead to an impairment of the enzyme activity.

p.H212N: although the H212 residue is exposed on the protein surface in the dimmer interface very close to the substrate binding site, the replacement with asparagine might be conservative as both residues, histidine (H) and asparagine (N), have a similar size, both are polar and protonated at acidic pH.

p.C309F: cysteina 309 is involved in a disulphide bond with its partner C360, which keeps two chains, A and B of β subunit tightly bound after the cleavage of the protein precursor. The mutation p.C309F would abrogate the formation of the disulphide bridge. However, hydrogen bond interactions, which occur among the strands within the protein beta-sheets, may hold chains A and B together, even in the absence of the disulphide bridge.p.G484E: this change, introducing a negatively charged glutamate (E), approximately 10A from the substrate binding site region, was predicted to interfere with the plasticity of the protein main chain and to alter the local electrostatic parameters.

Finally, structural analysis predicted that the p.R533C change, affecting a residue (R533) involved in the dimerization, might severely disturb the dimer formation. In addition, the novel C533 may form a disulphide bound with C551 (normally involved in a disulphide bound with C534), leading to a severe misfolding of the C terminal loop of the protein.

### Characterization of the Three Novel Splicing Mutations

The possible effect of the new intronic mutations c.1082+5G>A, c.1169+5G>A and c.1242+1G>A, on the mRNA splicing process was first evaluated *in silico* using two splicing prediction programs, NN splice and MES. Both programs clearly predicted that the mutated sequences would (i) no longer be recognized as splicing sites when the alteration involved one of the invariant motif of the classical consensus sequences (c.1242+1G>A) or (ii) cause a reduction in the strength of the splice site when mutations were located in non-canonically invariant splice site motifs (c.1082+5G>A and c.1169+5G>A) (Table 2).

**Table 2:** *In silico* analysis of novel splicing mutations.

Mutation	Prediction programs	
	NNWt score/mut score	MEWt score/mut score
**c.1082+5G>A**	5′ss: 8,85/3,23	5′ss: 0,91/0,26
**c.1169+5G>A**	5′ss: 9,72/4,06	5′ss: 0,94/0,14
**c.1242+1G>A**	5′ss: 9,60/1,42; 3′ss:1,32/1,32; Cryptic 3′ss: 6,49/6,49	5′ss: 1,00/ND; 3′ss: ND/ND;Cryptic 3′ss: 0,51/0,51

ND: Not detected; ME: Maximum Entropy; NN: Neural Network.

Since the fibroblasts from patients SD7, SD8, SD9, SD10 were not available, we used a minigene assay to study the effect of the c.1082+5G>A, c.1169+5G>A and c.1242+1G>A mutations on the mRNA splicing process. Normal and mutant minigenes were transfected into Hek293 cells and total RNAs were reverse transcribed to cDNAs. Specific PCR analysis showed the amplification of PCR products of different length in cells transfected with normal and mutant *HEXB* alleles. The identities of the PCR products were confirmed by sequencing.

**Figure 4:**
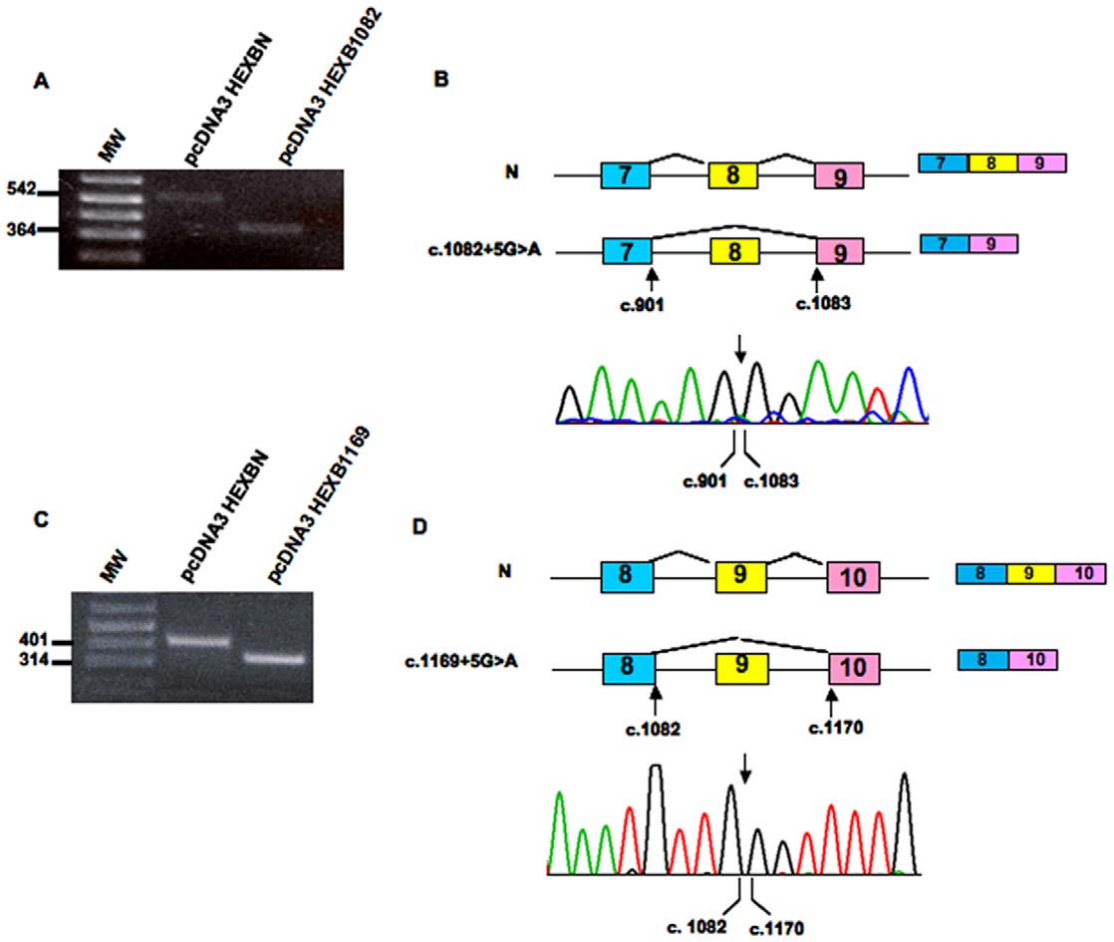
*In vitro* functional analysis of mutations c.1082+5G>A and c.1169+5G>A. RT-PCR analysis of the *HEXB* mRNA in cells transfected with normal (pcDNA3HEXBN) and minigenes containing mutations c.1082+5G>A (pcDNA3HEX1082) and c.1169+5G>A (pcDNA3HEX1169) (panels A and C, respectively). MW: 1 kb Plus DNA Ladder. Schematic representation of the effect of the novel mutations on the splicing process (panels B and D). Sequencing analysis of RT-PCR products showed that the c.1082+5G>A mutation determines the skipping of 178 nt of exon 8 (panel B), whereas the presence of c.1169+5G>A mutation determines the retention of 87 nt of exon 9 (panel D).

**Figure 5:**
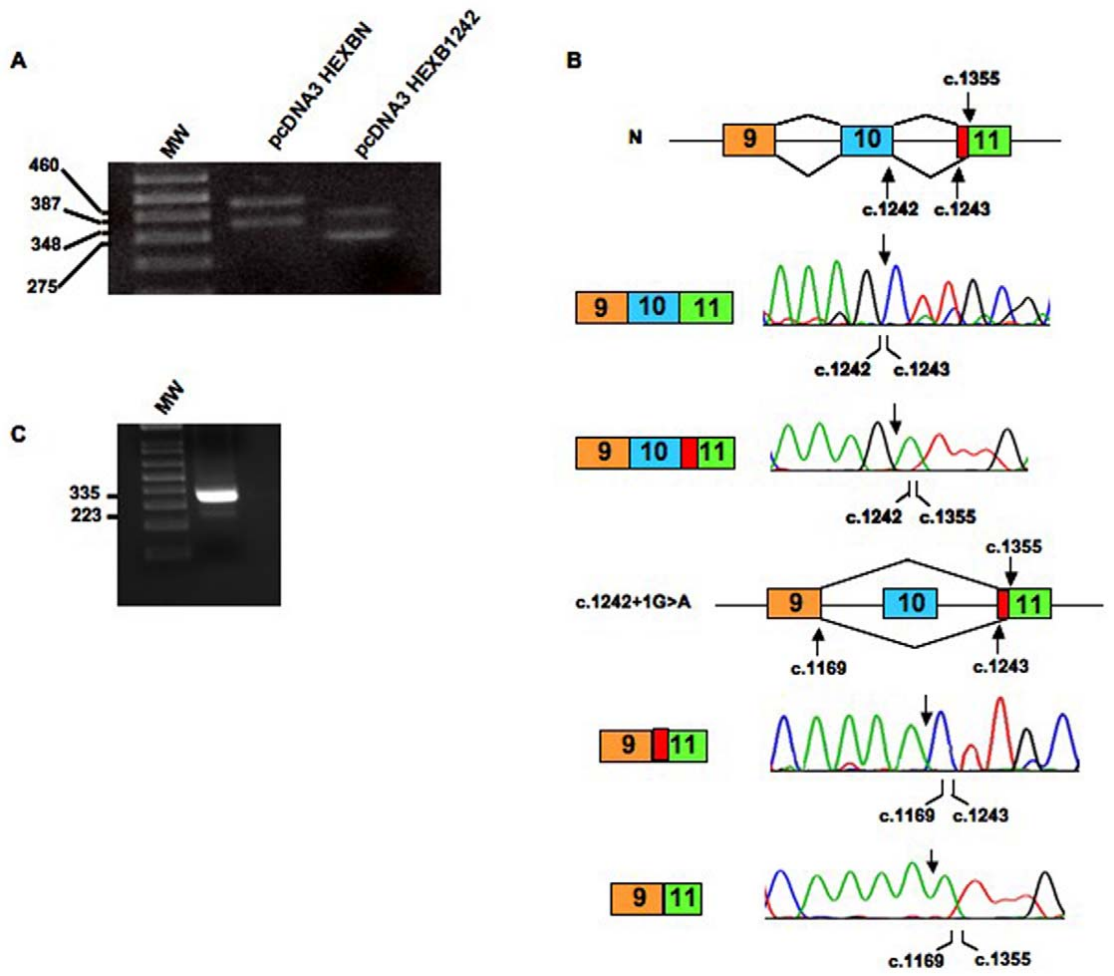
*In vitro* functional analysis of mutation c.1242+1G>A and exon 11 splicing analysis in normal fibroblasts. Panel A: RT-PCR analysis of the *HEXB* mRNA in cells transfected with normal (pcDNA3HEXBN) and a minigene containing mutation c.1242+1G>A (pcDNA3HEX1242) MW: 1 kb Plus DNA Ladder. Panel B: Schematic representation of the effect of the novel mutations on the splicing process. Sequencing analysis of RT-PCR products showed that the presence of c.1242+1G>A mutation determines the skipping of 73 nt of exon 10. In addition the presence of a 3′ cryptic splice site (present in both normal and mutant minigenes) determines the skipping of 112 nt in exon 11(denoted as red box) in cells transfected with both normal and mutant minigenes. Panel C: RT-PCR analysis of the *HEXB* mRNA in normal fibroblasts. MW: 1 kb Plus DNA Ladder.

As expected, the mutant construct bearing c.1082+5G>A produced a 181 nt shorter transcript, which lacks exon 8. The skipping of exon 8 would lead to the shifting in the reading frame and the generation of a premature stop codon (p.G301_W361delfsX10) (Figure 4a and b). In the same vein, the c.1169+5G>A mutation determined the predicted skipping of 87 nt of exon 9, causing the in-frame exclusion of 28 aminoacids from the mature protein (p.E362_K390del) (Figure 4c and d). The mutant c.1242+1G>A yielded two different transcripts (Figure 5a): i) a transcript lacking 73 nt corresponding to the entire sequence of exon 10, as predicted by *in silico* analysis, and ii) a transcript lacking 73 nt of exon 10 and a portion of exon 11 (Figure 5b). In fact, a cryptic splice site, located 112 nucleotides downstream of the normal acceptor site of exon 11, obtained a higher score than the canonical acceptor site when tested with both prediction programs used in this study (Table 2). The cryptic splice site was recognized as an acceptor splice site in cells transfected with the normal minigene construct as well (Figure 5a, b). The recognition of this cryptic splice site resulted in the exclusion of 112 nt from the mature transcript. In order to confirm this result *in vivo*, a RT-PCR analysis of the *HEXB* mRNA followed by sequencing of PCR products was performed on the RNA extracted from normal fibroblasts. Two mRNA variants were detected: one containing the entire exon 11 sequence and one lacking the 112 nt of exon 11. This result confirmed the data obtained by the minigene assay and indicates that two different *HEXB* transcripts, generated by alternative spicing, are present in normal cells (Fig. 5c).

## Discussion

Sandhoff disease is a severe neurodegenerative lysosomal storage disorder caused by mutations in the *HEXB* gene. To date, a total of 43 mutations of *HEXB* gene have been described as a cause of SD. In this study, we report the results from the molecular characterization of 14 SD unrelated patients with different geographical/ethnic background.

All patients presented with the severe infantile form of the disease except patient SD1 who had the subacute form of SD. In spite of this, high residual Hex activity was detected in the severely affected patients SD2, SD11 and SD14. Residual enzyme activity has been suggested to be related to clinical outcome in lysosomal diseases. However in these three patients phenotype and residual enzyme activity do not seem to correlate.

Several hypotheses could explain these results, including the concurrence of other genetic and epigenetic factors in influencing the phenotype [19]. Another underlying cause might be attributed to increased levels of Hex S, a third β-hexosaminidase isoenzyme composed of two α subunits (HexS), present in very small amounts in normal tissues. Indeed, increased levels of HexS have been previously reported as associated with the absence of beta subunits [20], [21]. Therefore, a small contribution of this isoenzyme to the residual activity detected in these patients cannot be excluded. In addition, it should be taken into account that the substrate for HexA *in vivo* is the GM2/GM2 activator complex [22]. Thus, it may also be possible that the mutations present in these patients lead to the synthesis of a HexA variant which cannot efficiently bind to the GM2 activator. In this case, the activity against the synthetic substrate may not reflect the activity *in vivo*. Finally, another factor to be considered is that variations in the levels of residual activity may be found in different tissues. Considering this hypothesis the clinical course of SD, characterized by a progressive neurodegeneration, may correlate with the enzymatic activity in the brain, which may differ from that detected in peripheral tissues.

In this study, 16 different mutations were identified; most of them were present in single or few families. The only exception was represented by the c.445+1G>A (r.0) mutation found in 4 out of 5 families of Argentinean origin. This mutation has already been described as the most frequent allele among Argentinean SD patients [15]. Notably, two Argentinean patients, SD7 and SD8, coming from a geographic area in Cordoba characterized by a high incidence of SD [15] carried the same genotype. As a close relationship was not proved between these families, the common origin from the same geographically localized area may explain this result.

During the course of the analysis of the SD4 patient’s family we noted that the results did not entirely concur among the family members. More specifically, while the p.T150P mutation was in apparent homozygosis in the patient, it was absent in one of his parents. Similarly, the second mutation in patients SD1 and SD3 remained uncharacterized after sequencing the entire coding region of *HEXB* gene. Therefore, among the possible underlying reasons for these findings, we hypothesized the presence of a deletion in the second allele. In fact, among the *HEXB* mutations already published, three large *HEXB* deletions have been identified: (i) a 16 kb deletion which removes the promoter region, the exons 1–5 and part of intron 5 [16], (ii) a 50 kb deletion which removes 25 kb 5′ of the promoter up to intron 6 [23] and (iii) a 2.5 Kb deletion which involves part of intron 1 and exon 2 [6]. In particular, the 16 kb deletion, accounting for 27% of the SD mutant alleles, has been reported as the most frequent mutation in patient affected from SD with different ethnic backgrounds [4], [5], but never identified in Argentinean [15] and Italian [6] populations.

Hence, we set up a Multiplex Ligation dependent Probe Amplification (MLPA) assay to ascertain accidental misgenotyping in our panel of SD patients and to analyze the alleles that remained uncharacterized after being studied by conventional techniques.

This experimental approach, based on the simultaneous determination of copy number variation in a semiquantitative fashion, was effective in identifying the common large deletion (16 kb) in an Italian SD patient for the first time and the rare c.299+1471_408del2406 mutation in another patient of Italian origin. One allele remained unknown even after MLPA analysis, suggesting the presence of a deep intronic mutation that may affect the mRNA splicing process. The analysis of the *HEXB* mRNA should be preformed to confirm this hypothesis.

These results confirmed by PCR and sequencing analysis, allowed us to validate the MLPA assay as a valuable, rapid and economic method for detecting large deletion in SD patients and to reduce the risk of misdiagnosis.

Overall, we identified 9 new sequence variations. Among them, the homozygous nonsense mutation c.146C>A (SD13) was predicted to introduce the premature stop codon likely leading to a very short truncated protein (p.S49X), while the always homozygous c.1260_1265delAGTTGA mutation (SD14) resulted in an in-frame deletion, which determined the absence of two conserved residues: V421 and E422 (http://www.ensembl.org/). In addition, structural analysis showed that the deletion of these two aminoacids within the T419-V423 beta-strand, would displace the position of the W424, which is important for the correct substrate orientation.

As report in Figure 2, *in vitro* functional analysis of the mutant p.T209I, p.G484E and R533C proteins, showing a very low or absent Hex activity, clearly demonstrated their pathogenic nature. The analysis of the possible effect of these residue changes on protein structure showed that these aminoacid replacements would cause a severe impairment of protein structure and function.

The functional analysis of proteins carrying the p.H212N and p.C309F aminoacid changes showed that both retained a quite high residual activity (Figure 2). Indeed, computational analysis predicted that the replacement of p.H212N must not lead to severe structural changes since both aminoacids, histidine (H) and asparagine (N) are similar in size and polarity and both are supposed to be protonated at acidic pH. Instead, the results of the functional and structural analyses did not concur in the case of the C309F mutation, as this residue change was predicted to abrogate the formation of the disulphide bridge that keeps two chains (A and B of β subunit) tightly bound after the cleavage of the protein precursor. Following these controversial results, a more detailed examination of the protein structure showed that hydrogen bond interactions, which occur among the strands within the protein beta-sheets, may hold chains A and B together. Therefore, it is likely that, a fraction of the expressed protein could still maintain a proper folding and be partially active thanks to the hydrogen bond interactions.

Unexpectedly, the high residual activity retained by proteins bearing the H212N and C309F changes did not correlate with the severe clinical phenotype presented by patients (SD11 and SD2). In particular, the p.H212N change, found at homozygosity in a severely affected patient born from consanguineous parents, caused only 41% reduction of Hex activity. Whether this mutation is in fact deleterious or causes a Hex pseudodeficiency remains unclear. However, it is worth to point out that the clinical features presented by the patient are strongly suggestive of a GM2 gangliosidosis and the sequence analysis of the *HEXA* and *GM2A* genes, showing the absence of mutations enabled us to exclude other clinical forms of GM2 gangliosidosis. In the light of these data, it is possible to hypothesize the occurrence of a second *in cis* mutation causing a severe impairment of mRNA processing and/or expression. Unfortunately, the patient’s mRNA was not available to test this hypothesis.

The mutation p.C309F, found in compound heterozygosity with the severe c.1303_1304delGT (p.R435fsX20) mutation, retained 36% of activity when expressed *in vitro*. In this case, the potential coexistence of other mutations on the same allele carrying the p.C309F was excluded by the analysis of the *HEXB* mRNA extracted from the patient’s fibroblasts. These findings suggest that factors other than the patients’ genotype might influence the severity of the disease. Discrepancies between the *in vitro* residual enzymatic activity and the clinical phenotype have been described in lysosomal storage disorders including SD [12], [24]–[27]. In particular, such miscorrelation has been reported by Yoshizawa et al [12] in a late onset SD phenotype found to be associated with to the HEXB mutant p.R533H, which was completely inactive when expressed *in vitro*.

Indeed, these discrepancies may be caused by the fact that the results obtained *in vitro* do not necessarily reflect in *vivo* alterations, when the mutated proteins are expressed at a certain level, in a particular cell type and organelle interacting with other proteins. However, for most enzymes it is impractical to measure the activity *in vivo* and in any case it is not possible to determine the effect of a single mutation in heterozygous patients. Therefore, we have to rely on *in vitro* assays, keeping in mind their limitations.

Finally, minigene studies performed to demonstrated the impact of the 3 new intronic mutations (c.1082+5G>A, c.1169+5G>A and c.1242+1G>A) upon the mRNA splicing process, revealed that a novel alternative spliced *HEXB* mRNA variant was also present in normal control cells, owing to the recognition of a cryptic splice site within exon 11. This finding suggests caution in the interpretation of results potentially leading to a molecular misdiagnosis.

In conclusion, our results confirmed the remarkable heterogeneity of the mutational spectrum of the *HEXB* gene and provided new insights into the molecular basis of SD. In addition, the present study stresses the importance of investigating copy number changes in order to avoid misinterpretation of the molecular testing results.

## Supporting Information

Table S1 **Probes and primers used for the MLPA assay.** a PCR product size (bp) b Universal primer sequences are in capital letters; gene target sequences are in small letters. *NC_000005.9 #NC_000004.11 §NC_000004.11 §NC_000010.10; stuffer sequences are in capital underlined letters.
(DOC)

Table S2 **Primers used for cloning and site directed mutagenesis.** RefSeq cDNA: NM_000521; RefSeq genomic: NC_000005.9.
(DOC)
