# Supplementary material for: Sequence and Copy Number Analyses of HEXB Gene in Patients Affected by Sandhoff Disease: Functional Characterization of 9 Novel Sequence Variants
Source: PLoS One. 2012 Jul 27;7(7):e41516. doi: 10.1371/journal.pone.0041516 (PMC3407239; doi:10.1371/journal.pone.0041516)
Supplement: Table S1 — Probes and primers used for the MLPA assay. a PCR product size (bp) b Universal primer sequences are in capital letters; gene target sequences are in small letters. *NC_000005.9 #NC_000004.11 §NC_000004.11 §NC_000010.10; stuffer sequences are in capital underlined letters. (DOC) [file pone.0041516.s001.doc]

**Table S1. Probes and primers used** for the MLPA assay

| **Probe** | **Size ^a^** | **fmol** | **Primer ^b^** | |
| --- | --- | --- | --- | --- |
| **HEXB*** |  |  |
| Exon 1 | 100 pb | 4 | 1FW | 5'-AGGTGCAACGAGGACGGACcggtgaagatgaccccgaacctgc-3' |
|  |  | 4 | 1RV | 5'-PHO-tgcatctcgccccggagaacttctacCCTTATCTAATTAGGACGAACGACCCAACCA-3' |
| Exon 2 | 103 pb | 4 | 2FW | 5'-AGGTGCAACGAGGACGGACcagatatcatggctatatttttggtttctacaagt-3' |
|  |  | 4 | 2RV | 5'-PHO-ggcatcatgaacctgctgaattccagAAAGAGGACGAACGACCCAACCA-3' |
| Exon 3 | 97 pb | 1 | 3FW | 5'-AGGTGCAACGAGGACGGACgtttggggagcattacgaggtaagttc-3' |
|  |  | 1 | 3RV | 5'-PHO-catgcagtttcattgttactttccagtaaaggAGGACGAACGACCCAACCA-3' |
| Exon 4 | 142 pb | 8 | 4FW | 5'-AGGTGCAACGAGGACGGACgaaacaacccccagggtatgggtacaatgaa-3' |
|  |  | 8 | 4RV | 5'-PHO-gtaaagcacttttattagatgagtagagaagtgcaatggGTTGCCCAACTTTACCCGCAATGCCCGCGCACATAGGACGAACGACCCAACCA-3' |
| Exon 5 | 107 pb | 4 | 5FW | 5'-AGGTGCAACGAGGACGGACgttggttactattgtgtagtttggaacaggggaa-3' |
|  |  | 4 | 5RV | 5'-PHO-tttgtataagcactgggcattctttcctcagttccAGGACGAACGACCCAACCA-3' |
| Exon 6 | 112 pb | 4 | 6FW | 5'-AGGTGCAACGAGGACGGACgtttgcttgcaggatgccatggctttta-3' |
|  |  | 4 | 6RV | 5'-PHO-ataagtttaatgttcttcactggcacatagttgatgacCGGATGCTAGGACGAACGACCCAACCA-3' |
| Exon 7 | 132 pb | 8 | 7FW | 5'-AGGTGCAACGAGGACGGACatggcttttacagggaagctattctttgtctc-3' |
|  |  | 8 | 7RV | 5'-PHO-atgtttatacaccaaatgatgtccgtatggtgattgAGGAAAGCAATACTCTGGGACACGTAAGGACGAACGACCCAACC-3' |
| Exon 8 | 125 pb | 4 | 8FW | 5'-AGGTGCAACGAGGACGGACgaaattagtgaggtgtttccagatcaattcattc-3' |
|  |  | 4 | 8RV | 5'-PHO- atttgggaggagatgaagtggaatttaaatgttgATTGTACTTCGGATGGGCCAGGACGAACGACCCAACCA-3' |
| Exon 9 | 128 pb | 4 | 9FW | 5'-AGGTGCAACGAGGACGGACcaagatttcatgaggcaaaaaggctttggc-3' |
|  |  | 4 | 9RV | 5'-PHO-acagattttaagaaactagaatctttctacattcaaaagtaagCCTTATCTGGTCATATTAGGACGAACGACCCAACCA-3' |
| Exon 10 | 121 pb | 4 | 10FW | 5'-AGGTGCAACGAGGACGGACggttttggatattattgcaaccataaacaagggat-3' |
|  |  | 4 | 10RV | 5'-PHO-ccattgtctggcaggaggtttttgatgAGGAAAGCAATACTCTGGGACAGGACGAACGACCCAACCA-3' |
| Exon 11 | 119 pb | 4 | 11FW | 5'-AGGTGCAACGAGGACGGACgctatggacaagattggaggaaatactataaagtg-3' |
|  |  | 4 | 11RV | 5'-PHO-gaacctcttgattttggcggtaagtgaagcCATTCTCTGGTTTTCGAGGACGAACGACCCAACCA-3' |
| Exon 12 | 138 pb | 8 | 12FW | 5'-AGGTGCAACGAGGACGGACggtactcagaaacagaaacaacttttcattggtg-3' |
|  |  | 8 | 12RV | 5'-PHO-tggagaagcttgtctatggggagaatatgCATTATCTGCAAATGTGAGGACGAACGACCCAACCA-3' |
| Exon 13 | 94 pb | 1 | 13FW | 5'-AGGTGCAACGAGGACGGACggcaagtgctgttggtgagagactct-3' |
|  |  | 1 | 13RV | 5'-PHO-ggagttccaaagatgtcagagatatggatgAGGACGAACGACCCAACCA-3' |
| Exon14 | 135 pb | 4 | 14FW | 5'-AGGTGCAACGAGGACGGACcaacctctttatgctggatattgtaaccatgag-3' |
|  |  | 4 | 14RV | 5'-PHO-aacatgtaaaaaatggaggggaaaaaggcacagCCGAATTTATCAGCTATGCAAAACATTGCGTAGGACGAACGACCCAACCA-3' |
| ALB# | 109 pb | 4 | ALB FW | 5’-AGGTGCAACGAGGACGGACcacagaatccttggtgaacaggcga-3’ |
|  |  | 4 | ALB RV | 5’-PHO-ccatgcttttcagctctggaagtcgatATTGTACTTCGGATGGGCCAGGACGAACGACCCAACCA-3’ |
| AFAP1§ | 105 pb | 4 | AFAP FW | 5'-AGGTGCAACGAGGACGGACggtacctacactaacacatgatgaaaacctc-3' |
|  |  | 4 | AFAP RV | 5'-PHO-aatatgtcctcaggtggtaccatgtaagcTAATTTAAGGACGAACGACCCAACCA-3' |
| ACTR1a^ | 115 pb | 8 | ACTR FW | 5'-AGGTGCAACGAGGACGGACggatgagttaattcacacagctttgtcagagc-3' |
|  |  | 8 | ACTR RV | 5'-PHO-cctcatgcagcctcttgtaagcagatagCATTCTCTGGTTTTCGAAGGACGAACGACCCAACCA-3' |

a PCR product size (bp)

b Universal primer sequences are in capital letters; gene target sequences are in small letters. *NC_000005.9 #NC_000004.11 §NC_000004.11 ^NC_000010.10; stuffer sequences are in capital underlined letters.
