# Supplementary material for: Sequence and Copy Number Analyses of HEXB Gene in Patients Affected by Sandhoff Disease: Functional Characterization of 9 Novel Sequence Variants
Source: PLoS One. 2012 Jul 27;7(7):e41516. doi: 10.1371/journal.pone.0041516 (PMC3407239; doi:10.1371/journal.pone.0041516)
Supplement: Table S2 — Primers used for cloning and site directed mutagenesis. RefSeq cDNA: NM_000521; RefSeq genomic: NC_000005.9. (DOC) [file pone.0041516.s002.doc]

**Table S2. Primers used for cloning and site directed mutagenesis**

| **Primer** | **Sequence*** |
| --- | --- |
| HEXB cDNA BamHI Fw2 | 5’-CGCGGATCCCGATGTTGGCGCTGCTGACTCAG-3’ |
| HEXB cDNA Xho Rv | 5’-CCGCTCGAGCATGTTCTCATGGTTACA-3’ |
| Minigene HEXB 7-8-9 Fw | 5’-ATAAGAATGCGGCCGCATGGGAAGCTATTCTTTGTCTCAT-3’ |
| Minigene HEXB 7-8-9 Rv | 5’-CCGCTCGAGTTTTGAATGTAGAAAGATTCTAG-3’ |
| Minigene HEXB 8-9-10 Fw | 5’-ATAAGAATGCGGCCGCATGGTCAGAAAGACCTCCT-3’ |
| Minigene HEXB 8-9-10 Rv | 5’-CCGCTCGAGCTTTGCTTTATCATCAAAAACCTC-3’ |
| Minigene HEXB 9-10-11 Fw | 5’-ATAAGAATGCGGCCGCATGGAATCAAATCCAAAAATTCAA-3’ |
| Minigene HEXB 9-10-11 Rv | 5’-CCGCTCGAGCGCCAAAATCAAGAGGTTCCAC-3’ |
| HEXB T209I Fw | 5’-ACAGAGGAATTTTGATTGATATATCCAGACATTATCTGACAG-3’ |
| HEXB T209I Rv | 5’-CTGGCAGATAATGTCTGGATATATCAATCAAAATTCCTCTGT-3’ |
| HEXB H212N Fw | 5’-GGAATTTTGATTGATACATCCAGAAATTATCTGCCAGTTAAGATT-3’ |
| HEXB H212N Rv | 5’-AATCTTAACTGGCAGATAATTTCTGGATGTATCAATCAAAATTCC-3’ |
| HEXB C309F Fw | 5’-GAAAGACCTCCTGACTCCATTTTACAGTAGACAAAACAAG-3’ |
| HEXB C309F Rv | 5’-CTTGTTTTGTCTACTGTAAAATGGAGTCAGGAGGTCTTTG-3’ |
| HEXB G484E Fw | 5’-GAAACAACTTTTCATTGGTGAAGAAGCTTGTCTATGGGGA-3’ |
| HEXB G484E Rv | 5’-TCCCCATAGACAAGCTTCTTCACCAATGAAAAGTTCTTTC-3’ |
| HEXB R533C Fw | 5’-ATGACAGACTGACAAGGCACTGCTGCAGGATGGTCGAAC-3’ |
| HEXB R533C Rv | 5’-GTTCGACCATCCTGCAGCAGTGCCTTGTCAGTCTGTCAT-3’ |
| HEXB 1082+5G>A Fw | 5’-GAAGTGGAATTTAAATGTTGGTAAATGATTCCTTAAAACCCCTT-3’ |
| HEXB 1082+5G>A Rv | 5’-AAGGGGTTTTAAGGAATCATTTACCAACATTTAAATTCCACTTC-3’ |
| HEXB 1169+5G>A Fw | 5’-GAATCTTTCTACATTCAAAAGTAAATTGTTTGAAAGCCTATTTCTG-3’ |
| HEXB 1169+5G>A Fw | 5’-CAGAAATAGGCTTTCAAACAATTTACTTTTGAATGTAGAAAGATTC-3’ |
| HEXB 1242+1G>A Fw | 5’-GGTTTTTGATGATAAAGCAAAGATGAGCATTGTGAAGACTGC-3’ |
| HEXB 1242+1G>A Rv | 5’-GCAGTCTTCACAATGCTCATCTTTGCTTTATCATCAAAAACC-3’ |

RefSeq cDNA: NM_000521; RefSeq genomic: NC_000005.9
